# Propolis mouthwashes efficacy in managing gingivitis and periodontitis: a systematic review of the latest findings

**DOI:** 10.1038/s41405-025-00294-z

**Published:** 2025-01-25

**Authors:** Muhammed Al-Huda Ballouk, Mohamed Altinawi, Abeer Al-Kafri, Talar S. Zeitounlouian, Piotr S. Fudalej

**Affiliations:** 1 Department of Paediatric Dentistry, Faculty of Dentistry, Damascus University, Damascus, Syria; 2 Department of Lab Medicine, Faculty of Medicine, Damascus University, Damascus, Syria; 3 Department of Orthodontics, Faculty of Dentistry, Damascus University, Damascus, Syria; 4 Department of Orthodontics, Institute of Dentistry, Medical Faculty, Jagiellonian University, Kraków, Poland; 5 Department of Orthodontics, Institute of Dentistry and Oral Sciences, Faculty of Medicine and Dentistry, Palacký University Olomouc, Olomouc, Czech Republic; 6 Department of Orthodontics, School of Dental Medicine, Medical Faculty, University of Bern, Bern, Switzerland

**Keywords:** Gum disease, Periodontics, Preventive dentistry

## Abstract

**Background and objectives:**

Gingivitis and periodontitis are common periodontal diseases that can significantly harm overall oral health, affecting the teeth and their supporting tissues, along with the surrounding anatomical structures, and if left untreated, leading to the total destruction of the alveolar bone and the connective tissues, tooth loss, and other more serious systemic health issues. Numerous studies have shown that propolis can help reduce gum inflammation, inhibit the growth of pathogenic bacteria, and promote tissue regeneration, but with varying degrees of success reported. For this reason, this comprehensive systematic review aims at finding out the truth concerning the efficacy of propolis mouthwashes in treating gingivitis and periodontitis, as its main objective.

**Data sources:**

Research findings from 6 different databases: China National Knowledge Infrastructure (CNKI), PubMed®, Europe PMC, Cochrane Central Register of Controlled Trials (CENTRAL), BioMed Central, and Google Scholar, were retrieved and examined in addition to a manual search in the references lists.

**Study selection and synthesis:**

The PICOS framework was used to select and exclude studies. The focus was on clinical randomized controlled trials (RCTs) that examined the effectiveness of propolis-containing mouthwashes in comparison with propolis-free ones for the treatment of gingivitis and periodontitis, employing related periodontal indices. Animal studies, microbiological studies, in-vitro studies, retrospective studies, case-control studies, cohorts, case reports, case series, reviews, letters, editorials, meta-analyses, and non-clinical randomized controlled trials (non-RCTs), all were excluded. A meta-analysis was not performed and data were only studied qualitatively due to the obvious heterogeneity amongst the studies. Data from the selected studies were extracted, and then the revised Cochrane’s risk of bias tool (RoB 2.0) was utilised by two of the authors, independently, to evaluate the risk of bias in each study.

**Results:**

At first, 151 results were reached, but then after removing duplicates, 99 records remained, and were later screened, assessed, and studied in full details based on the set PICOS criteria. Out of these 99 articles, ten studies were included in this systematic review, encompassing a total of 453 patients with an age range of (13–70) years old. Propolis mouthwashes with different protocols of application were the intervention whereas placebo or the rest of the tested mouthwashes such as, chlorhexidine, sodium fluoride with cetylpyridinium chloride, sterile distilled water, hydrogen peroxide, were the ones to which propolis mouthwashes were compared. Treatment duration extended from 14 days to 3 months and the follow-up period differed from 14 days to 3 months. In general, propolis mouthwashes decreased plaque accumulations and gingival inflammation in gingivitis patients based on the employed indices. On the other hand, the aforementioned tested mouthwashes other than propolis were deemed equally effective or even superior to propolis in some studies. As an overall assessment for the risk of bias, four studies were assigned as having a low risk of bias. Two studies were deemed to have some concerns, while four studies were identified as having a high risk of bias.

**Conclusions:**

Despite the fact that propolis has shown positive effects in terms of controlling gingival and periodontal inflammation especially when used with mechanical methods, studies lack certainty and their power of evidence is low with no agreed gold standards. These conclusions come, for sure, within the limitations of this review, like having substantial variability amongst the included studies and the presence of studies with a high risk of bias. The findings demonstrate that propolis-based mouthwashes showed promising clinical outcomes in reducing plaque and gingival inflammation. However, it is highly recommended to conduct more rigorous trials with patient-reported outcome measures, extended follow-up periods, larger samples sizes, better-designed methodologies, typified propolis use, and with the implementation of similar indices in order to obtain more reliable, conclusive, and generalisable results.

**Prospero Registration Number:**

CRD42024524523.

## Introduction

It is unquestionable that oral healthcare is an important factor that positively contributes to the overall quality of life and general health [1]. Therefore, guaranteeing adequate oral hygiene, including all dental, gingival, and periodontal aspects, is a matter of great importance that can never be overestimated [2]. Insufficient oral care leads to many bad health sequelae, amongst which dental caries and gingivitis rank first and second [3, 4].

Gingivitis is a widely-prevalent concern in oral health for children and adolescents [5], arising from a variety of factors but primarily linked to inadequate oral hygiene practices [6]. It can be associated with serious systemic diseases like cardiovascular diseases, diabetes mellitus, osteoporosis, respiratory diseases, premature birth, and oral cancer [7]. Its presence is marked by observable clinical signs including inflammation, discomfort, and marginal bleeding [8], all of which contribute to have a negative impact on the oral health-related quality of life (OHRQoL) [9, 10]. It is essential to effectively manage gingivitis to prevent further damage to the underlying tissues and potential tooth loss, which is known as periodontitis [11, 12]. Periodontitis refers to a series of irreversible long-lasting infectious and inflammatory conditions that can harm the supporting tissues of the teeth, including the periodontal ligament, alveolar bone, and root cementum [13]. It is the major cause of tooth loss [14]. This condition is a significant public health concern, affecting more than half of the global adult population, and its prevalence tends to increase with age [15, 16] whereas severe periodontitis affects 11% of the world population [17]. Various methods, both surgical and non-surgical, have been employed to address and deal with periodontitis, with a preference of non-invasive approaches. However, the limitations of relying solely on scaling and root planning sometimes necessitate the use of antimicrobial agents to decrease the presence of harmful bacteria in the gingiva and underneath the gingival margin [18]. All the aforementioned information emphasizes the critical role of achieving optimal oral hygiene, that is second to none in the treatment of gingivitis and periodontitis, which involves a bundle of procedures like regular toothbrushing, flossing, and employing interdental brushes or even the newly mechanical methods such as powered flossing, end-tufted brushes, and oral irrigation devices [19, 20]. However, it is important to note that depending upon mechanical plaque removal through these methods might not be entirely sufficient, highlighting the use of adjuncts and innovative methods for better outcomes [21]. One of these very important adjuncts is the use of mouthwashes. Mouthwashes are particularly valuable in this regard, as they aid in controlling dental plaque in hard-to-reach areas and offer convenience in application [22]. Mechanical plaque removal through brushing is an irreplaceable gem in the chain of keeping optimum oral health, however, mouthwashes, give an additional shine and support via a plaque disruption mechanism besides their ease of access and use, since they require neither good dexterity nor an additional instrument in order to apply them. Consequently, the use of mouthwashes, especially those containing herbal or natural ingredients like propolis, has garnered significant attention in recent studies due to their positive effects in maintaining good oral health [23–25].

Propolis is an age-old substance that has been utilized extensively throughout human history and continues to have applications in mostly all medical domains, and for both therapeutic and preventive purposes [26, 27]. Nowadays, researchers are uncovering its immense advantages, exploring the potential of propolis in uncharted areas and its role as a fundamental or ancillary treatment for certain illnesses [28, 29]. In one interesting concise narrative review, the authors list many uses and applications for propolis, presenting over 100 studies in all the different specialties of dentistry and oral medicine, with both preventive and therapeutic modes of action [30]. As a result, and that being said, propolis emerges as an exceptionally promising material that justifies additional scrutiny, particularly due to its natural origins that offer fewer or even no negative consequences when compared to the synthetic substances currently employed that have indisputable and inevitable adverse effects not to mention the high expenses associated with synthetic pharmaceuticals [31–33]. These facts push clinicians to move towards more natural, secure, and cost-effective alternatives [34].

To explore this material further, propolis is a resinous substance produced by honey bees to protect their hives from invaders and infections [35, 36]. It contains over 300 different components and possesses numerous health benefits [37, 38]. These include antioxidant, antimicrobial, antibacterial, anti-inflammatory, anticancer, analgesic, antidepressant, and immunomodulant effects [39–42]. Hence, propolis offers a wide range of active clinical applications even medically. It has shown promising results and can also aid in the management of many persistent medical conditions like diabetic foot ulcers as well as its potential in helping cure many types of cancer [43–45]. In addition to that, the flavonoids found in propolis are believed to be responsible for these positive effects besides many more favourable ones [46, 47].

Once again, in the oral arena, propolis provides distinct advantages. For example, its antifungal properties yield comparable outcomes to those of miconazole [48]. Propolis has proven successful in treating recurrent aphthous ulcers, reducing halitosis, and efficiently combating oral herpes infections [49–51]. It has been shown to eliminate even the pathogenic and opportunistic microbes with a good cariostatic effect [52]. When it comes to endodontic procedures, propolis stimulates the regeneration of dentin in cases of direct pulp capping with similar or superior properties to those of calcium hydroxide [53, 54]. Orthodontic patients and those with dental implants have shown a decrease in gingival inflammation [55, 56], thanks to the antibacterial and anti-inflammatory properties of propolis. This reduction in inflammation has also led to a decrease in plaque-induced gingivitis [57]. Furthermore, the phenols and flavonoids present in propolis help inhibit periodontal pathogens that contribute to periodontal pocket depth and bleeding on probing [58].

In a 2020 systematic review [59], the authors focused on the effects of propolis in reducing symptoms of gingivitis, alone, when compared to chlorhexidine only. The resulting evidence was weak and further more robust studies, especially RCTs, with larger sample sizes were recommended. In the scientific literature, there have been limited RCT investigations into the efficacy of propolis mouthwashes in treating periodontitis [60]. Therefore, this systematic review was undertaken to critically and systematically appraise the existing evidence, unravel the misconceptions regarding the true clinical effectiveness of propolis-based mouthwashes as adjuncts to traditional oral hygiene practices for reducing both gingivitis and periodontitis, as well as report any potential side effects observed in recent years. This systematic review was prompted by the availability of new evidence concerning propolis mouthwashes, highlighting the need for a comprehensive evaluation [61–65]. In simpler wording, this present systematic review aims at finding out the reality as to the effectiveness of propolis mouthwashes in the course of treating gingivitis and periodontitis.

## Materials and methods

The protocol was registered in the PROSPERO database (CRD42024524523).

This systematic review was written according to the Cochrane Handbook for Systematic Reviews of Interventions 2^nd^ edition [66] and the Preferred Reporting Items for Systematic Reviews and Meta-Analyses (PRISMA) guidelines [67].

### Eligibility criteria

The **PICOS** framework was used to select and exclude studies as follows:

### P. Participants

#### Inclusion criteria

The included studies were Clinical Randomized Controlled Trials (RCTs) that have recruited patients experiencing symptoms of gingivitis or periodontitis. There were no restrictions based on age, sex, ethnicity, or socioeconomic status in our systematic review.

#### Exclusion criteria

Animal studies, microbiological studies, and those investigating the effects of propolis mouthwashes in patients with dental removable prostheses, orthodontic appliances, or systemic diseases, all were excluded.

### I. Intervention

#### Inclusion criteria

This systematic review encompassed studies in which patients have received any treatment protocols involving propolis-based mouthwashes (i.e., propolis as the sole active ingredient in the mouthwashes), irrespective of the specific dosage, concentration of the active substance, application timing, duration, frequency, or follow-up.

#### Exclusion Criteria

Studies incorporating other adjuncts along with or prior to the propolis mouthwash application (e.g., electric toothbrushes) and which could help control plaque accumulation (rather than conventional methods) were also excluded due to the possibility of masking the sole effects of mouthwashes and their ability to eliminate gingivitis or periodontitis. Studies in which the applied experimental propolis mouthwashes had other active ingredients along with propolis (e.g., herbal extracts in addition to propolis) were excluded, too.

### C. Comparison

#### Inclusion criteria

Comparison groups of included studies consisted of any participants who were exposed to non-propolis-based mouthwashes, placebo mouthwashes, or did not use any mouthwashes at all. Each and every study can depend on different gold standards based on some special scientific rationale, and when this justifying reasoning was provided, the comparison was deemed to be valid.

#### Exclusion criteria

Studies where the comparisons were inappropriate, such as those comparing different approaches or means of application that were incompatible and non-harmonious (e.g., comparing mouthwashes to water jets or any other mechanical plaque removal methods), were excluded.

### O. Outcomes

#### Inclusion criteria

The assessment was focused on evaluating indices related to gingivitis (gingival inflammation) and periodontitis (periodontal inflammation), including the Plaque Index of Sillness and Löe (PI), Approximal Plaque Index of Lange (API), O’Leary Plaque Control Record Index (OPI), Turesky-Modified Quigley-Hein Plaque Index (TQHPI), Oral Hygiene Index (OHI), Oral Hygiene Index-Simplified (OHI-S), Patient Hygiene Performance Index (PHP), Gingival Index of Löe and Sillness (GI), Modified Gingival Index (MGI), Bleeding on Probing (BOP), Papillary Bleeding Index (PBI), Gingival Bleeding Index (GBI), Periodontal Probing Depth (PPD), Clinical Attachment Level (CAL), Community Periodontal Index of Treatment Needs (CPITN), and Community Periodontal Index (CPI). These are the indices most related to the conditions which were under consideration. Additionally, any other related parameters, indices, or even side effects were also looked at and considered, when relevant.

#### Exclusion criteria

Studies with only DMFT, dmft, DMFS, dmfs, or any other indices which have nothing to do with the gingival and periodontal aspect of the oral health status were excluded.

### S. Study Design

#### Inclusion criteria

Only human randomized clinical controlled trials (RCTs) were considered for inclusion in this systematic review.

#### Exclusion criteria

Animal studies, in vitro studies, retrospective studies, case-control studies, cohorts, case reports, case series, reviews, letters, editorials, and meta-analyses were all excluded.

### Search strategy

Multiple databases were used to conduct the electronic search: China National Knowledge Infrastructure (CNKI), PubMed®, Europe PMC, Cochrane Central Register of Controlled Trials (CENTRAL), BioMed Central, and Google Scholar, from January 2013 to April 2024, by two independent reviewers (MAB and TSZ). Table 1 provides more information about the keywords used and the search strategy. Additionally, other potentially related articles listed in the reference lists, which may not have been obtained through the electronic search, were also included.

**Table 1:** The detailed electronic search strategy.

**CNKI**	
Publication date:	(Gingivitis + Gingival Inflammation + Gingival Disease + Periodontitis + Periodontal Inflammation + Periodontal Disease)
From January 2013 to April 2024	
Search builder:	AND (Mouthwashes + Mouthrinses + Mouthwash + Mouthrinse) AND (Propolis + Bee Glue)
Title, Keyword & Abstract	
**PubMed**	
Publication date:	(Gingivitis[Title/Abstract] OR Gingival Inflammation[Title/Abstract] OR Gingival Disease[Title/Abstract] OR Periodontitis[Title/Abstract] OR Periodontal Inflammation[Title/Abstract] OR Periodontal Disease[Title/Abstract]) AND (Mouthwashes[Title/Abstract] OR Mouthrinses[Title/Abstract] OR Mouthwash[Title/Abstract] OR Mouthrinse[Title/Abstract]) AND (Propolis[Title/Abstract] OR Bee Glue[Title/Abstract])
From January 2013 to April 2024	
Search builder:	
Title/Abstract	
**Europe PMC**	
Publication date:	(ABSTRACT:“propolis” OR TITLE:“propolis” OR ABSTRACT:“bee glue” OR TITLE:“bee glue”) AND (ABSTRACT:“mouthwashes” OR TITLE:“mouthwashes” OR ABSTRACT:“mouthwash” OR TITLE:“mouthwash” OR ABSTRACT:“mouthrinses” OR TITLE:“mouthrinses” OR ABSTRACT:“mouthrinse” OR TITLE:“mouthrinse”) AND (ABSTRACT:“gingivitis” OR TITLE:“gingivitis” OR ABSTRACT:“gingival inflammation” OR TITLE:“gingival inflammation OR ABSTRACT:“gingival disease” OR TITLE:“gingival disease “ OR ABSTRACT:“periodontitis” OR TITLE:“periodontitis” OR ABSTRACT:“periodontal inflammation” OR TITLE:“periodontal inflammation” OR ABSTRACT:“periodontal disease” OR TITLE:“periodontal disease”)
From January 2013 to	
April 2024	
Search builder:	
Title OR Abstract	
**CENTRAL**	
Publication date:	(“propolis” OR “bee glue”):ti,ab,kw AND (“mouthwashes” OR “mouthwash” OR “mouthrinses” OR “mouthrinse”):ti,ab,kw AND (“gingivitis” OR “gingival inflammation” OR “gingival disease” OR “periodontitis” OR “periodontal inflammation” OR “periodontal disease”):ti,ab,kw
From January 2013 to April 2024	
Search builder:	
Title/Abstract/Keyword	
**BioMed Central**	
Publication date:	(Propolis “OR” bee glue) AND (Mouthwashes “OR” Mouthrinses “OR” Mouthwash “OR” Mouthrinse) AND (Gingivitis “OR” gingival inflammation “OR” Gingival Disease “OR” periodontitis “OR” Periodontal Inflammation “OR” Periodontal Disease)
From January 2013 to April 2024	
**Google Scholar**	
Publication date:	(Propolis “OR” bee glue) AND (Mouthwashes “OR” Mouthrinses “OR” Mouthwash “OR” Mouthrinse) AND (Gingivitis “OR” gingival inflammation “OR” Gingival Disease “OR” periodontitis “OR” Periodontal Inflammation “OR” Periodontal Disease)
From January 2013 to April 2024	

### Study selection, data extraction and synthesis

Two reviewers perused the study selection individually (MAB and TSZ), disagreements or conflicting results were resolved by other reviewers (MA and PSF) through consensus. Titles and abstracts were screened then full text articles were retrieved to include the related ones based on the determined criteria. General information (authors’ names, publication date), study design, sample size, mean age, interventions (application of mouthwashes and comparisons), application protocol, evaluation time, clinical parameters and assigned teeth, adverse events and results (PI, API, OPI, TQHPI, OHI, OHI-S, PHP, GI, MGI, BOP, PBI, GBI, PPD, CAL, CPITN, CPI) were the extracted characteristics from the assigned articles. A qualitative synthesis was used for various outcome measures across the included studies. However, due to the obvious heterogeneity amongst the studies, a quantitative synthesis was not performed.

### Risk of bias assessment

The revised Cochrane risk of bias tool for RCTs (RoB 2.0) [68] was utilized to assess the risk of bias in the included studies by two independent reviewers (MAB and TSZ) as a judgement (high, low, and some concerns). Other reviewers (MA and PSF) were consulted in case of any disagreements with the results. The tool evaluated bias in five domains including randomization, deviations from intended interventions, missing outcome data, measurement bias, and selection bias in reported results. The risk of bias evaluation was based on the most critical evaluation. When all fields were found to have a low risk of bias, the overall risk of bias was considered low. If one or more fields were discovered to have some concerns of bias, they were classified as having some concerns about bias. On the other hand, if one or more fields were evaluated as having a high risk of bias, it was assumed to have a high risk of bias in general.

## Results

### Study selection

**Fig. 1:**
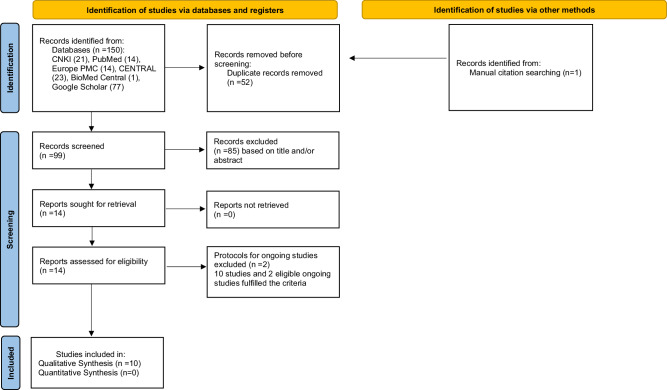
The PRISMA flow-chart. Diagram of the reviewing process, study selection, and inclusion.

A thorough electronic search was conducted across multiple databases, including China National Knowledge Infrastructure (CNKI), PubMed®, Europe PMC, Cochrane Central Register of Controlled Trials (CENTRAL), BioMed Central, and Google Scholar, which yielded a total of 150 articles. Additionally, we manually searched the references of the selected articles and found one more relevant article. An overlap was seen among databases. Therefore, 52 duplicate articles were removed and then the remaining 99 studies were screened for their eligibility. Any articles that did not meet the selection criteria were excluded from further analysis. Based on titles and abstracts, 85 articles were excluded. After carefully examining the full texts of 10 trials along with 4 protocols of ongoing studies, 2 protocols were excluded with the reasons provided in Table 2. As a result, 10 trials and 2 ongoing studies were included in our systematic review. Figure 1 presents the PRISMA flow diagram illustrating the screening and inclusion processes.

**Table 2:** The excluded protocols.

Study ID and Title	Reason for Exclusion
ACTRN12622000215729 *(Date Registered: the 7*^*th*^ *of February, 2022)* A randomized, double-blind, non-inferiority trial evaluating the efficacy and safety of two different mouthrinses on patients with gingival inflammation. https://www.anzctr.org.au/Trial/Registration/TrialReview.aspx?id=382522&isReview=true	Comparing a polyherbal mouthwash containing propolis to CHX and CITROX® (formulation deriving from the extract of bitter orange peel) (Curaprox Italia S.r.l., Bolzano, Italy).
IRCT20220717055482N1 *(Date Registered: the 18*^*th*^ *of June, 2023)* Effect of the mouthwash containing propolis-aloe vera plant. https://trialsearch.who.int/Trial2.aspx?TrialID=IRCT20220717055482N1	The mouthwash is not solely composed of propolis but rather combined with aloe vera.

### General characteristics of the included studies

The relevant information from the assigned 10 articles was extracted and summarized in Table 3. However, the characteristics of the 2 ongoing studies can be found in Table 4. The overall analysis included a total of ten randomized clinical trials (RCTs) involving 453 patients, whose ages ranged from 13 to 70. The sample size of the study participants varied from 28 to 120 individuals. Sex information was reported in over half of the studies [62, 64, 69–72], with males comprising 8 to 27 and females comprising 11 to 36 depending on each individual study, for the remaining studies did not mention age distribution. The studies were carried out in various locations, with four studies taking place in India [71–74], three in Brazil [69, 70, 75], two in Iran [62, 63], and one in Malaysia [64]. The proposed sample sizes of the two uncompleted studies which are based in Iran, are 40 and 78 with the inclusion of both sexes. All of the included studies involved participants who were generally in good health. Additionally, any medications that could potentially affect the intervention being studied were excluded. The observation period ranged from fourteen days to three months.

**Table 3:** Characteristics of the included studies.

Authors, Publication Year, and Country	Study Design	Sample Size, (Male/Female), and Mean Age	Intervention, Protocol of Application, and Control	Propolis Recipe	Observation Period	Clinical Parameters and Assigned Teeth	Results and Adverse Events
**Anauate-Netto et al**. **(2014)** [69] **Brazil**	RCT 3-arm parallel group	60 patients, (24/36), and (18–55 y)	G1: (*n* = 20) 2% propolis G2: (*n* = 20) 0.12% CHX G3: (*n* = 20) placebo 15 ml of mouthwashes twice daily after conventional oral hygiene practices	Alcohol-free, 2% typified propolis, mint flavour, water, polioxyethelers, sorbitol, and blue colour. Propolis rinse was manufactured at the laboratories of the Department of Pharmacology at Federal University of Santa Catarina, Florianopolis, SC, Brazil	At baseline and 28 days	PBS on the mesio-buccal surfaces of all teeth	Reduction in PBI/PBS in propolis group was superior to all groups and significant when compared to 0.12% CHX. Adverse effects were detected in all groups with the least number of reports (*n* = 7) presented in propolis (breath alteration, burning sensation, yellow teeth, taste alteration, and bitter taste) when compared to CHX (*n* = 23) and placebo (*n* = 9)
**Bretz et al**. **(2014)** [70] **Brazil**	RCT (co-twin) study design	38 patients (16/22) and (13–22 y) with induced gingivitis G1: (16.1 ± 2.59 y) G2: (16.1 ± 2.59 y) with induced gingivitis following oral hygiene promotion phase	G1: (*n* = 19) 2% propolis G2: (*n* = 19) 0.05% sodium fluoride NaF + 0.05% cetylpyridinium chloride CPC mouthwashes 25 ml for 30 sec twice daily	2% typified propolis, mint flavour, polioxyethelers, sorbitol, blue colouring, and water. Propolis rinse was made by using green propolis (State of Minas Gerais, Brazil)	At -14 days (baseline), and 0 (after hygiene phase) and 21 days (no hygiene phase)	PBS on the mesial buccal surfaces of all teeth Average gingival redness (G- parameter) of the anterior dentition	No significant differences between both groups in terms of PBS and G-parameter. Tooth discoloration was detected in one patient.
**Santiago et al**. **(2018)** [75] **Brazil**	RCT 4-arm parallel group	40 patients, (20–40 y)	G1: (*n* = 10) placebo (sterile distilled water) G2: (*n* = 10) 2.6% propolis G3: (*n* = 10) 0.12% CHX G4: (*n* = 10) 0.06% CHX with 1.3% propolis 15 ml of mouthwashes for 1 min once daily	Propolis was collected in the Beekeeping Section, UNESP, Campus of Botucatu. Ethanolic extracts of propolis (30%) were prepared (30 g of propolis, completing the volume to 100 mL with 70% ethanol)	At baseline and 14 days	PHP in the vestibular surfaces of the upper right first molar and upper right central incisor, in the lingual surface of the first lower left molar, in the labial surface of the lower left central incisor, and in the lingual surface of the lower right first molar	Both chlorhexidine and propolis revealed comparable results
**Porwal et al**. **(2018)** [73] **India**	RCT 3-arm parallel group	30 patients, (20–40 y), with chronic generalized gingivitis	G1: (*n* = 10) 0.2% CHX diluted with distilled water (1:1) G2: (*n* = 10) raw propolis diluted with distilled water (1:1) G3: (*n* = 10) 3% hydrogen peroxide 10 ml of mouthwashes twice daily	Not available	At baseline, 7 days, and 28 days	TQHPI and MGI	Comparable results were obtained with the dominance of CHX regarding plaque control. However, propolis was more effective in reducing gingival index
**Krishna et al**. **(2019)** [71] **India**	RCT 3-arm parallel group	45 patients (27/18) and (18–70 y) with chronic generalized gingivitis	G1: (*n* = 15) 5% propolis G2: (*n* = 15) 0.2% CHX G3: (*n* = 15) saline (placebo) 10 ml of mouthwashes 4 times daily twice in the morning and twice at night	Not available	At baseline and 6 weeks	PI and GI	Propolis was superior to CHX in terms of plaque accumulation and gingival inflammation control
**Hongal et al**. **(2019)** [72] **India**	RCT 3-arm parallel group	30 patients (10/20) and (18–60 y) with chronic generalized gingivitis	G1: (*n* = 10) propolis G2: (*n* = 10) CHX G3: (*n* = 10) saline (placebo) for 1 min twice daily	Not available	At baseline and 14 days	PI and GI	Chlorhexidine was the most effective in controlling gingivitis whereas no significant differences were found regarding plaque control
**Bapat et al**. **(2021)** [74] **India**	RCT 4-arm parallel group	120 patients and a mean age of (18–22 y)	G1: (*n* = 30) hot ethanolic propolis extract G2: (*n* = 30) cold ethanolic propolis extract G3: (*n* = 30) 0.2% CHX G4: (*n* = 30) distilled water (placebo) 10 ml of mouthwashes for 1 min twice daily	Hot ethanolic propolis: By dissolving raw propolis into 70% ethyl alcohol. Propolis (100 gm) was mixed with 1000 ml of solvent ethyl alcohol (99.97 grade) by hot continuous method to yield 5 g of semisolid extract. This extract was further diluted to a concentration of 5 μg/ml. Cold ethanolic propolis: By dissolving raw propolis into 70% ethyl alcohol. Propolis (100 g) was mixed with 1000 ml of solvent ethyl alcohol (99.97 grade) at room temperature and further diluted to a concentration of 5 μg/ml	At baseline, 15 days, I month, and 3 months.	PI and GI of four surfaces (buccal, lingual, mesial, and distal) of all teeth	No significant differences were found in plaque and gingival scores between propolis groups and CHX group at all time intervals meaning that propolis is as effective as CHX in reducing plaque and gingivitis. No adverse effects (allergic reactions or burning sensation) were observed among participants of all groups.
**Kiani et al**. **(2022)** [62] **Iran**	RCT 2-arm parallel group	32 patients (8/24) and (19–55 y) G1: (31.5 ± 10.15 y) G2: (33.25 ± 6.99 y) with gingivitis	G1: (*n* = 16) propolis G2: (*n* = 16) non-propolis 30 drops of mouthwashes were dissolved in 20 ml of water for 1 min twice daily	Propolis (®Impident, Gyahan Sabz Zendegy Co.Iran, Tehran)	At baseline, 15, and 30 days on the tooth with the most inflamed gingiva in each quadrant	PBI PI Gingival Modification of the stain index. All teeth were evaluated and the measurements of the tooth with the most inflamed gingiva in each quadrant were recorded.	Reduction in papillary bleeding was significantly greater in propolis group unlike PI that showed no significant differences between both groups except for the two-week period that showed reduction of PI in favour of propolis group. Insignificant tooth staining was seen in propolis, whereas it was significant in the placebo. Side effects such as gingival redness, mucositis, and tooth or mucosal discoloration were not seen in any case. Nothing was seen other than some insignificant staining.
**Salari et al**. **(2023)** [63] **Iran**	RCT 2-arm parallel group	28 patients (18–50 y) with chronic generalized gingivitis	G1: (*n* = 14) CHX 0.2% G2: (*n* = 14) propolis 30% 10 ml of mouthwashes for 60 sec twice daily	Not available	At baseline and 2 weeks	OPI, PI, and GI of the whole oral cavity	CHX was significantly more effective than propolis in plaque control, whereas propolis was similar to CHX in reducing GI. Propolis is a natural and safe alternative for CHX with no side effects for daily use.
**Gunjal and Pateel** **(2024)** [64] **Malaysia**	RCT Latin-square, cross-over study design	45 patients (16/29) and (18–30 y) with chronic generalized gingivitis	G1: (*n* = 15) propolis 0.2% G2: (*n* = 15) CHX 0.12% G3: (*n* = 15) placebo mouthwashes 10 ml (undiluted) for 1 min twice daily	5% propolis, mint flavour, propylene glycol, sorbitol, and water. Propolis rinse was made by using propolis from Malaysia (NHF, Malaysia).	At baseline and 21 days	PI and GI (full mouth)	Propolis showed a statistically significant reduction in both PI and GI when compared to CHX.

**Table 4:** Characteristics of the ongoing studies.

Study ID	Country	Title	Study Design	Age/Gender	Intervention, Protocol of Application, and Control	Outcomes
**IRCT20180528039880N1**	Iran	Comparison of the Effectiveness of Propolis and Chlorhexidine Mouthwash on Gingival Inflammation in Chronic Gingivitis	Randomized double-blinded parallel RCT	No limit/both	Group 1: propolis mouthwash Group 2: CHX 0.12% mouthwash 15 cc for 1 min twice daily for 10 days	PI, GI, and CPI
**IRCT20180130038558N1**	Iran	Evaluation of the Effectiveness of Mucoadhesive Mouthwash Containing Propolis Extract Formulation on Prevention and Improvement of Symptoms in Patients with Generalized Chronic Gingivitis	Randomized double-blinded parallal RCT	18–60 years/both	Group 1: 5% propolis mouthwash Group 2: CHX mouthwash Group 3: placebo mouthwash For 30 sec twice daily for 14 days	BOP, GBI, MGI, and TQHPI

### Intervention and comparison groups

Propolis mouthwashes were used in all of the studies included in this review. The rinsing duration varied, with most studies implementing a twice-daily rinsing protocol [62–64, 69, 70, 72–74]. However, the frequency of rinsing was once and four times daily in the studies [71, 75] respectively. The effect of propolis was assessed using different formulations. Two studies [69, 70] used a 2% propolis formulation, 2.6% propolis was used in Santiago et al.’s study [75], 5% propolis in Krishna et al.’s study [71], Salari et al. [63] applied 30% propolis and Gunjal and Pateel [64] used 0.2% propolis formulations with unclear information about propolis concentration was provided in three studies [62, 72, 73]. Cold and hot propolis extracts were used in one study [74]. The comparison was made against either a placebo or other mouthwashes such as chlorhexidine gluconate [63, 64, 69, 71–75]. 0.12% chlorhexidine was adopted in Anauate et al.’s study [69]. On the contrary, many studies [63, 64, 71, 73, 74] used a concentration of 0.2% chlorhexidine, whereas 0.12% and 0.06% chlorhexidine with 1.3% propolis served as two independent control groups in Santiago et al.’s study [75]. Hongal et al. provided no information about chlorhexidine’s concentration [72]. Both sodium fluoride NaF and 0.05% cetylpyridinium chloride (CPC) were implemented in Bretz et al.’s study [70]. A non-propolis mouthwash was employed in Kiani et al.’s study [62]. Chlorhexidine was the mouthwash to which propolis is compared in the two incomplete studies.

### Outcome measures

There was a significant variation in the reporting of outcomes across the studies as shown in Table 5. When it comes to documenting plaque, different indices were utilised: five studies [62, 64, 71, 72, 74] employed the PI, whereas Santiago et al.’s study adopted the PHP [75], Salari et al.’s [63] used the OPI, and the TQHPI was used in Porwal et al.’s study [73]. In terms of assessing gingival status, most of the studies [63, 64, 71, 72, 74] employed the GI, while Bretz et al.’s study [70] used average gingival redness (G parameter) and another study employed MGI [73], also gingival modification of the stain index was the measurement used in Kiani et al.’s research [62]. PBS/PBI was implemented to observe bleeding associated with gingival inflammation in three studies [62, 69, 70]. As to the indices mentioned in the protocols of the two ongoing studies: GI, PI, CPI, BOP, GBI, MGI, and TQHPI.

**Table 5:** Means/Medians/Frequencies and standard deviations of the used indices in the included studies.

Authors (Year)	Propolis Group			Control Group		
	Index	Baseline	After Treatment	Baseline	After Treatment	*P*-value
Anauate-Netto et al. 2014 [69]	PBS	1.0(0.5)	0.5(0.5)	1.1(0.5)	0.9(0.6)	<0.05 **
Bretz et al. 2014 [70]	PBS	0.29(0.32)	0.51(0.48)	0.37(0.43)	0.48(0.39)	>0.05
	G-parameter	95(17.87)	91.2(16.60)	96.8(12.61)	94.9(14.05)	>0.05
Santiago et al. 2018 [75]	PHP	2.6(2.8)	2.0(2.2)	3.2(3.4)	2.0(2.2) *	N/A
Porwal et al. 2018 [73]	TQHPI	3.34(0.25)	0.94(0.57)	3.26(0.53)	0.82(0.45)	0.721
	MGI	3.16(0.34)	0.28(0.25)	3.04(0.23)	0.54(0.35)	0.225
Krishna et al. 2019 [71]	PI	1.95(0.07)	1.47(0.21)	1.94(0.08)	1.61(0.16)	0.04 **
	GI	1.86(0.19)	1.42(0.2)	1.86(0.19)	1.53(0.14)	0.21
Hongal et al. 2019 [72]	PI	1.29(0.32) #	0.11(0.68) #	1.33(0.20) #	0.27(0.24) #	*P* > 0.05
	GI	1.56(0.11) #	1.06(0.30) #	1.67(0.18) #	0.64(0.65) #	*P* < 0.05 **
Bapat et al. 2021 [74]	PI	0.98(0.10)	0.47(0.22)	1.04(0.05)	0.45(0.10)	*P* > 0.05
	GI	1.12(0.85)	0.43(0.09)	1.10(0.08)	0.46(0.06)	*P* > 0.05
Kiani et al. 2022 [62]	PI (*n* = 128 teeth)					*P* = 0.45
	0	27(42) ##	57(89.0) ##	37(57.9) ##	58(90.6) ##	
	1	11(18) ##	5(7.8) ##	13(20.3) ##	3(4.7) ##	
	2	24(37) ##	1(1.6) ##	13(20.3) ##	3(4.7) ##	
	3	2(3) ##	1(1.6) ##	1(1.5) ##	0(0) ##	
	PBI (*n* = 128 teeth)					*P* < 0.001 **
	0	2(3.1) ##	42(65.62) ##	0(0) ##	7(10.93) ##	
	1	9(14.07) ##	14(23.43) ##	4(6.25) ##	17(26.56) ##	
	2	44(68.76) ##	6(9.37) ##	51(79.68) ##	32(50) ##	
	3	9(14.07) ##	1(1.56) ##	9(14.06) ##	8(12.5) ##	
Salari et al. 2023 [63]	OPI	92.5(6.85)	33.21(5.96)	90.64(8.65)	21.71(1.63)	*P* = 0.00 **
	GI	1.57(0.75)	0.35(0.49)	1.42(0.85)	0.21 (0.42)	*P* = 0.1
Gunjal and Pateel 2024 [64]	PI	1.37(0.27)	0.7(0.25)	1.35(0.28)	0.90(0.17)	*P* < 0.001 **
	GI	1.31(0.24)	0.65(0.26)	1.30(0.25)	0.90(0.20)	*P* < 0.001 **

*the mean is missing; the authors only reported the range of means;

# median;

## number of teeth (%);

** statistically significant difference.

### Main outcomes

Out of eight studies that investigated plaque accumulation [62–64, 71–75], two studies [64, 71] supported the superior effectiveness of propolis in controlling plaque. Conversely, Kiani et al.’s study [62] found no significant differences between propolis and non-propolis mouthwashes in reducing dental plaque at all time points except for the second week as propolis showed better results. Similarly, four studies [72–75] reported that propolis was equally effective as chlorhexidine in controlling plaque, while Salari et al.’s study [63] reported that chlorhexidine mouthwashes were superior to propolis (Table 3). Regarding the gingival inflammation, three studies [64, 71, 73] favored the effect of propolis over other mouthwashes in reducing the gingival index (GI). However, two studies [63, 74] did not find propolis to be superior to chlorhexidine in improving gingival health. Moreover, Hongal et al.’s study [72] favoured the effect of chlorhexidine over propolis in terms of the gingival situation. When it comes to reducing the PBI values, better findings were observed with propolis [62, 69] in contrast to the findings of Bretz et al.’s trial [70] that found comparable results between propolis and sodium fluoride NaF with cetylpyridinium chloride (CPC) regarding PBI and G-parameter (Tables 3 and 5).

### Adverse effects

Not all the included studies provided information on the potential side effects associated with the use of propolis mouthwashes [64, 71–73, 75] However, in one study [69], it was found that 7 patients experienced a breath alteration, burning sensation, yellow teeth, taste alteration, and a bitter taste when compared to 23 in the chlorhexidine group and 9 in the placebo group, whereas only one patient had slight tooth discoloration in another study [70]. No significant side effects such as gingival redness, or mucositis were detected in any of the cases and only insignificant staining was observed in Kiani et al.’s study [62]. However, two studies [63, 74] demonstrated no negative or detrimental effects resulting from the use of propolis.

### Risk of bias of the included studies

Table 6 summarizes the risk of bias of the included studies with detailed justifications for each domain. In total and as an overall assessment for risk of bias, four studies [62, 64, 69, 70] were assigned as having a low risk of bias. Two studies [72, 74] were deemed to have some concerns, while four studies [63, 71, 73, 75] were identified as having a high risk of bias. In terms of potential bias during the randomization process, five studies [63, 71–73, 75] raised some concerns or were deemed to have a high risk. Additionally, five studies [63, 71–73, 75] were judged to have an uncertain risk of bias because they did not adequately blind the participants and personnel involved. When it comes to bias resulting from the blinding of individuals assessing the outcomes, most studies [63, 71–75] had some concerns or were at high risk in this aspect. It’s important to note that with regards to bias due to missing outcome data and selection of the reported result, all studies except [75] had a low risk of bias. It [75] was judged to have some concerns in the missing outcome data domain and a high risk of bias in the selection of the reported result domain.

**Table 6:** The risk of bias assessment of the retrieved studies according to RoB 2.0 tool 2019.

Author/Year	Bias Arising from the Randomization Process	Bias due to Deviations from Intended Interventions	Bias due to Missing Outcome Data	Bias in Measurement of the Outcome	Bias in Selection of the Reported Result	Overall Bias
**Anauate-Netto et al**. **2014** [69]	Authors’ judgement: Low risk Support for judgement: “A computer-generated list of random numbers was used. Rinses were prepared in dark bottles which were consecutively numbered according to the randomization schedule. Participants were randomized to one of the three test color-matched rinses. Study coordinator, examiners, and participants were unaware of group allocation”.	Authors’ judgement: Low risk Support for judgement: “Study coordinator, examiners, and participants were unaware of group allocation”.	Authors’ judgement: Low risk Support for judgement: All outcome data were available	Authors’ judgement: Low risk Support for judgement: “Study coordinator, examiners, and participants were unaware of group allocation”.	Authors’ judgement: Low risk Support for judgement: The measurements of outcomes described in the methods section were all reported.	Authors’ judgement: Low risk
**Bretz et al**. **2014** [70]	Authors’ judgement: Low risk Support for judgement: “A computer-generated list of random numbers to allocate groups. Rinses were prepared in dark bottles, which were consecutively numbered according to the randomization schedule. Twins were randomly assigned to one of the two test color-matched rinses. The study coordinator, examiners, and participants were unaware of group allocation”.	Authors’ judgement: Low risk Support for judgement: “The study coordinator, examiners, and participants were unaware of group allocation”.	Authors’ judgement: Low risk Support for judgement: Almost all outcome data were available.	Authors’ judgement: Low risk Support for judgement: “The study coordinator, examiners, and participants were unaware of group allocation”.	Authors’ judgement: Low risk Support for judgement: The measurements of outcomes described in the methods section were all reported.	Authors’ judgement: Low risk
**Santiago et al**. **2018** [75]	Authors’ judgement: Some concerns Support for judgement: The randomization method along with allocation concealment were not mentioned	Authors’ judgement: Some concerns Support for judgement: There is no information about this.	Authors’ judgement: Some concerns Support for judgement: The availability of all outcome data was not mentioned.	Authors’ judgement: High risk Support for judgement: There is no information about this.	Authors’ judgement: High risk Support for judgement: No means and *p*-values for PHP were mentioned.	Authors’ judgement: High risk
**Porwal et al**. **2018** [73]	Authors’ judgement: Some concerns Support for judgement: “The subjects were randomly divided into three equal groups”. The allocation concealment was not mentioned.	Authors’ judgement: Some concerns Support for judgement: There is no information about this.	Authors’ judgement: Low risk Support for judgement: All outcome data were available.	Authors’ judgement: High risk Support for judgement: There is no information about this.	Authors’ judgement: Low risk Support for judgement: The measurements of outcomes described in the methods section were all reported.	Authors’ judgement: High risk
**Krishna et al**. **2019** [71]	Authors’ judgement: Some concerns Support for judgement: “A total of 45 randomly selected patients (lottery method)”. The allocation concealment was not mentioned.	Authors’ judgement: Some concerns Support for judgement: There is no information about this.	Authors’ judgement: Low risk Support for judgement: “All the patients who were selected for the study completed the study protocol”.	Authors’ judgement: High risk Support for judgement: There is no information about this.	Authors’ judgement: Low risk Support for judgement: The measurements of outcomes described in the methods section were all reported.	Authors’ judgement: High risk
**Hongal et al**. **2019** [72]	Authors’ judgement: Some concerns Support for judgement: “Participants were randomly divided into 3 groups”. The allocation concealment was not mentioned	Authors’ judgement: Some concerns Support for judgement: “The clinician was blinded”.	Authors’ judgement: Low risk Support for judgement: All outcome data were available.	Authors’ judgement: Some concerns Support for judgement: There is no information about this.	Authors’ judgement: Low risk Support for judgement: The measurements of outcomes described in the methods section were all reported.	Authors’ judgement: Some concerns
**Bapat et al**. **2021** [74]	Authors’ judgement: Low risk Support for judgement: “Subjects were randomly allocated by lottery method into 4 study groups. Each participant was asked to take a slip from a box containing 120 slips with equal number of four different codes for the products and they were allotted to that group. The subjects were designated to the groups by a person not involved in the examination”.	Authors’ judgement: Low risk Support for judgement: “All the preparations were made to look alike and were delivered to the participants in similar bottles marked “A”, “B” or “C”.	Authors’ judgement: Low risk Support for judgement: All outcome data were available.	Authors’ judgement: Some concerns Support for judgement: Although the outcomes assessors could have been blinded, the blinding of outcomes assessment was not obviously mentioned.	Authors’ judgement: Low risk Support for judgement: The measurements of outcomes described in the methods section were all reported.	Authors’ judgement: Some concerns
**Kiani et al**. **2022** [62]	Authors’ judgement: Low risk Support for judgement: “The patients were randomly divided into two groups of propolis mouthwash and the placebo mouthwash using a table of random numbers”. “The mouthwash bottles were coded and the participants enrolled in the study by an assistant not involved in the study”.	Authors’ judgement: Low risk Support for judgement: “The dentist who was trained for this program- prescribing the mouthwashes, was blinded to the type of mouthwash used. The mouthwashes were prepackaged in bottles, then according to a randomization chart the bottles numbered for each patient. Patients received the mouthwashes in corresponding bottles”.	Authors’ judgement: Low risk Support for judgement: All outcome data were available.	Authors’ judgement: Low risk Support for judgement: All clinical examinations were performed by a trained general dentist under the supervision of a periodontist”. “The examiner was blinded to the type of mouthwash used”	Authors’ judgement: Low risk Support for judgement: The measurements of outcomes described in the methods section were all reported.	Authors’ judgement: Low risk
**Salari et al**. **2023** [63]	Authors’ judgement: Some concerns Support for judgement: “Patients were enrolled using a simple sampling method by gradual referrals and randomly assigned to groups”. The allocation concealment was not mentioned.	Authors’ judgement: Some concerns Support for judgement: “Each patient was given a similar bottle of mouthwash without a label”.	Authors’ judgement: Low risk Support for judgement: All outcome data were available.	Authors’ judgement: High risk Support for judgement: There is no information about this.	Authors’ judgement: Low risk Support for judgement: The measurements of outcomes described in the methods section were all reported.	Authors’ judgement: High risk
**Gunjal and Pateel** **2024** [64]	Authors’ judgement: Low risk Support for judgement: “The use of a computer-generated random allocation sequence, the random allocation was concealed by having a person not involved in the study”	Authors’ judgement: Low risk Support for judgement: “Each group was exposed to all three interventions in a phased manner (block randomization)”.	Authors’ judgement: Low risk Support for judgement: All outcome data were available.	Authors’ judgement: Low risk Support for judgement: “The clinician who performed all measurements was blinded to the treatment arms to the patients”	Authors’ judgement: Low risk Support for judgement: The measurements of outcomes described in the methods section were all reported.	Authors’ judgement: Low risk

## Discussion

Plenty are the factors related to gingivitis and periodontitis. For example, inadequate maintenance of oral hygiene is a contributing factor that causes an imbalance in the microorganisms residing in the mouth, known as the oral flora, sometimes called the oral microbiome. This imbalance, referred to as dysbiosis [76], can lead to the development of oral diseases, just like gingivitis, due to an excessive growth of harmful bacteria, forming advanced bacterial communities known as a biofilm [77]. To address dysbiosis and the formation of biofilms, it is recommended to reduce or eliminate the population of these bacteria in the mouth using mechanical and chemical methods, such as regular brushing with a toothpaste, and using mouthwashes [78, 79] that are deemed to be less dexterity or technically sensitive when compared to mechanical methods [80].

Out of all bacterial-control agents, propolis has gained recognition for its remarkable capacity to prevent oral diseases [81], along with its role in eliminating and inhibiting the formation of biofilms [82, 83], besides being a natural product, of course. This is particularly important since the main cause of both gingivitis and periodontitis is the formation of bacterial biofilms, with specific bacteria such as *Streptococcus Mutans, Porphyromonas Gingivalis, Tannerella Forsythia*, and *Treponema Denticola* being highly pathogenic [84–86]. Propolis has been extensively studied and found to offer numerous advantages in various areas of health, including overall well-being and oral health. Propolis has been applied in the field of periodontology [87], oral medicine [88], oral surgery [89], orthodontics [90], endodontics [91], prosthodontics [92], and restorative dentistry [93]. This systematic review specifically examined the effectiveness of propolis mouthwashes which are considered to be cost-effective, user-friendly, and generally associated with fewer adverse effects compared to the widely used chlorhexidine mouthwashes which can cause discoloration to the teeth, dorsum of the tongue, and dental restorations as well as altered and bitter taste in the mouth, and sometimes swelling of the parotid gland, hence, limiting their long-term use [94–96]. As a result, propolis has emerged as an alternative to these synthetic mouthwashes for managing gingivitis, which is a reversible condition that can be prevented by adopting optimal oral hygiene practices [76]. Notably, this systematic review is the first to assess the role of propolis mouthwashes in controlling plaque, gingival inflammation, and periodontitis, all together combined, and comparing propolis mouthwashes to various commonly used mouthwashes, rather than solely focusing on chlorhexidine only. Furthermore, the review did not impose any age restrictions.

This review extensively examined the effectiveness of propolis-based mouthwashes in controlling plaque. The results demonstrated that propolis exhibited a comparable antiplaque effect, if not superior in some studies, to the widely recognized gold standard, chlorhexidine mouthwashes. These antibacterial properties can be attributed to the presence of flavonoids, aromatic acid and esters in resins [88, 97, 98]. Propolis possesses the ability to impede colonization, growth, and metabolic processes of bacteria, thereby disrupting the development of bad mature biofilms and causing good favourable alterations at both the biochemical and ecological levels [35]. Furthermore, its antimicrobial properties in specific have been found to provide protection against dental plaque, too, for it was found that propolis could effectively combat a wide range of oral microorganisms [35] besides its ability to create calcium phosphates on the tooth surface contributing to the prevention of dental plaque formation by a second route [99].

Propolis has demonstrated remarkable results in improving gingival health. Numerous studies have confirmed that propolis mouthwashes are efficient and not inferior to other mouthwashes in reducing both the GI and PBI. In other words, propolis has shown more promising results than other mouthwashes [62, 64, 69, 71, 73], and some depicted similar results of propolis when compared to the most commonly used synthetic mouthwashes [63, 70, 74]. One of the important and notable reasons for its effectiveness is its ability to inhibit the production of prostaglandins, which play a key role in inflammatory responses, pain, and tissue inflammation. Propolis achieves this by inhibiting the enzymes lipoxygenase and cyclooxygenase, resulting in a rapid decrease in tissue inflammation [100]. In addition, an element called caffeic acid phenethyl ester CAPE found in propolis has revealed anti-gingivitis properties, highlighting its anti-inflammatory characteristics [101]. Moreover, flavonoids possess both direct and indirect antioxidant properties, which respectively include the ability to scavenge free radicals and induce the production of natural antioxidant enzymes within the body. Flavonoids are associated with antimicrobial, antioxidant, and anti-inflammatory effects [102].

Although propolis has shown pronounced outcomes with minimal side effects in the included studies focusing on gingivitis control, it is essential to approach its effectiveness with caution. Therefore, it was crucial to implement the revised Cochrane risk of bias tool RoB 2.0 to evaluate the strength of the included studies. This tool analyses various factors that can affect the study’s quality, ranging from biases that may arise during the randomization process to biases in the selection and reporting of results. Many studies were considered low-quality, resulting in inconclusive findings. In various regions around the world, propolis compositions generally share common fundamental components but also exhibit differences [103]. Additionally, the methodological approaches and outcomes varied significantly, making it challenging to perform a meta-analysis and obtain pooled data.

Periodontitis is a serious global health burden, ranking as the 11^th^ most prevalent disease worldwide [104], as dental caries and periodontitis are the two most common oral cavity diseases [105]. It is mainly promoted by bacteria in dental plaque, which generate pro-inflammatory substances and activate the local immune response, resulting in damage to the supportive tissues of the teeth and gradual loss of bone and attachment structures [106]. Even though the clinical research that was examined for this systematic review did not meet the specified inclusion criteria, it is important to note that certain studies have provided evidence suggesting that the use of propolis-based products can lead to a reduction in the PPD. This decrease is attributed to the propolis’ capacity to inhibit inflammatory processes and impede the growth of microorganisms within the periodontal pockets [62, 107, 108]. Furthermore, research has indicated that propolis has the ability to stimulate fibroblast activity and promote collagen synthesis, which contributes to tissue regeneration processes and ultimately leads to an improvement in the CAL [107, 109]. BOP is an important clinical measure used to assess the condition of the periodontal tissues and determine their inflammatory status. The findings of several studies revealed the effectiveness of propolis in reducing bleeding during periodontitis treatment, thanks to its antimicrobial, anti-inflammatory, and antioxidant properties, which play a significant role in the healing process following mechanical periodontal therapy [65, 110]. Nonetheless, the lack of evidence highlights the need for further investigations in this specific field.

According to the American Dental Association ADA [111], the recommended observation period for any antiplaque or anti-gingivitis agents, is at least six months. However, the follow-up periods of all the included studies were: two weeks [63, 72, 75], three weeks [64, 70], four weeks [62, 69, 73], six weeks [71], and the longest one was found in Bapat et al.’s study [74], which lasted three months. None of these periods meet the ADA’s requirements and are insufficient for assessing the true clinical effectiveness of the evaluated mouthwashes. This represents the main limitation of the retrieved studies. However, this can be justified by the need to avoid the unfavourable or side effects of some of the mouthwashes, like those occurring when using chlorhexidine for long durations. Other limitations include the inclusion of patients from different age groups with inconsistent recording of their sex, variations in sample size ranging from 28 to 120, as well as differences in propolis compositions, maybe, leading to variations in the proposed methodology. Also, the overall achieved quality varied between studies.

## Conclusions

The findings of this systematic review demonstrate that propolis-based mouthwashes showed promising clinical outcomes in reducing plaque and gingival inflammation. However, due to the substantial variability amongst the included studies and the presence of studies with a high risk of bias, it is highly recommended to conduct more rigorous trials with patient-reported outcome measures, extended follow-up periods, larger samples sizes, better-designed methodologies, typified propolis use, and with the implementation of similar indices in order to obtain more reliable, conclusive, and generalisable results which can then provide a strong scientific basis for recommending the use of propolis-based mouthwashes. Furthermore, such positive recommendations can encourage the healthcare sectors to incorporate this natural treatment option into their national and international programmes.

## Supplementary information

PRISMA Table

## Data Availability

All data generated or analysed during this study are included in this published article.
